# Supplementary material for: A magnetic hydrogel for the efficient retrieval of kidney stone fragments during ureteroscopy
Source: Nat Commun. 2023 Jun 22;14:3711. doi: 10.1038/s41467-023-38936-1 (PMC10287666; doi:10.1038/s41467-023-38936-1)
Supplement: Supplementary file 1 — Supplementary Information [file 41467_2023_38936_MOESM1_ESM.pdf]

Supplemental Figures

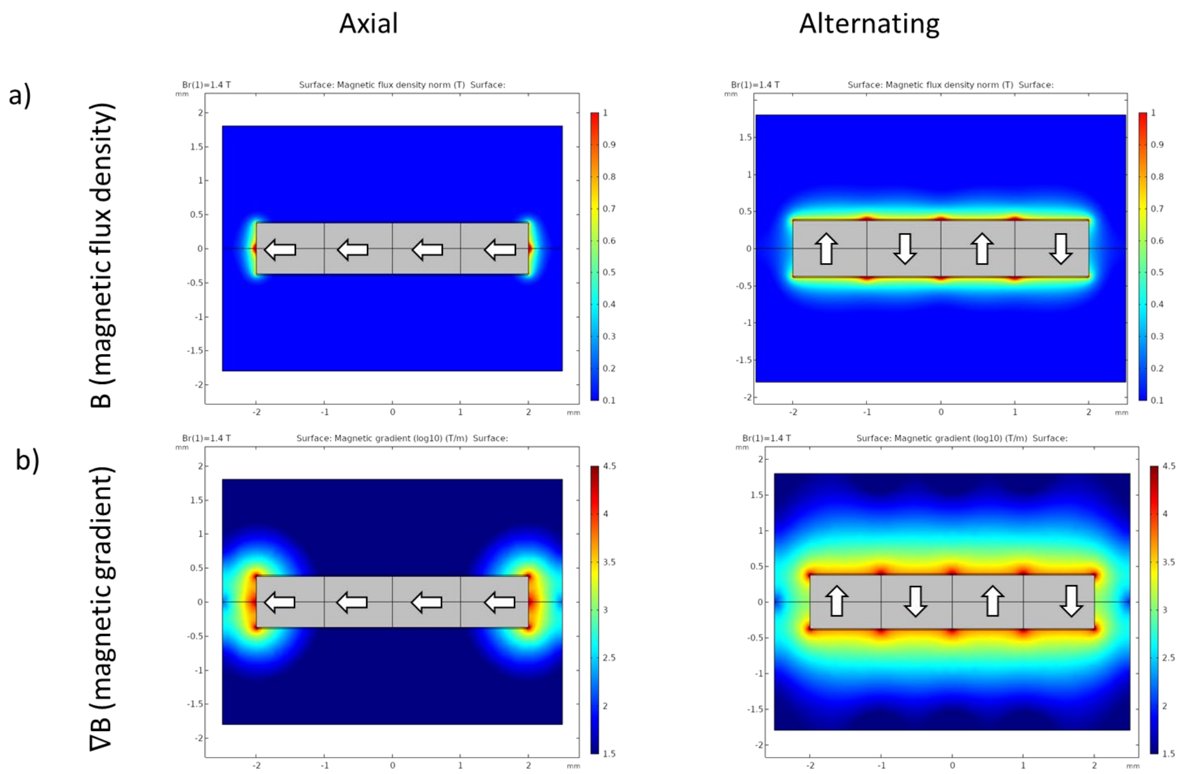

Figure S1. **a)** Simulated magnetic flux density (T) of magnets with unidirectional axial polarities (left) versus alternating diametric polarities (right). **b)** Simulated magnetic gradients (T/m) of magnets with unidirectional axial polarities (left) versus alternating diametric polarities (right). White arrows point to the north pole of the magnet.

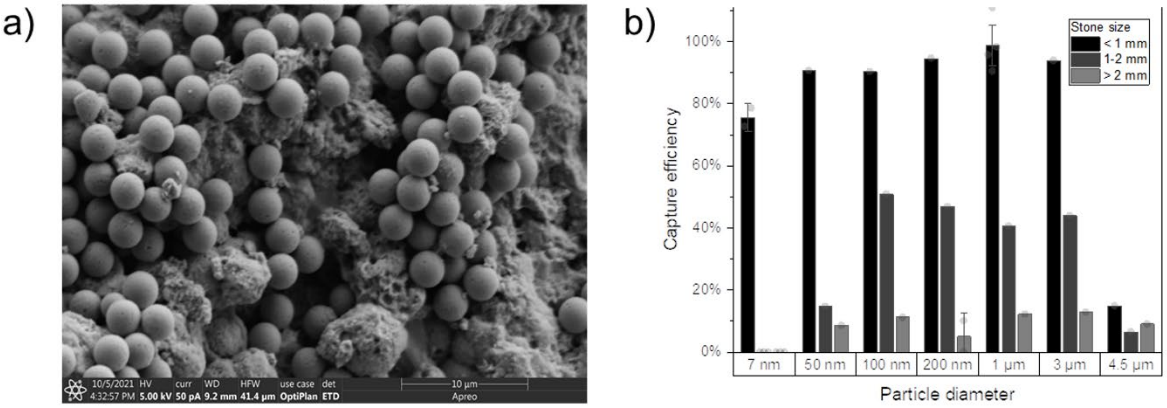

Figure S2. **a)** SEM image of 100% CaP stone coated with 3 micron diameter superparamagnetic iron oxide beads. n = 1-4 independent experiments for each group. Bars represent 1.5 standard error. **b)** Capture efficiency of SPIONs and larger superparamagnetic iron oxide beads for 100% CaP stones of different sizes. Source data are provided as a Source Data file.

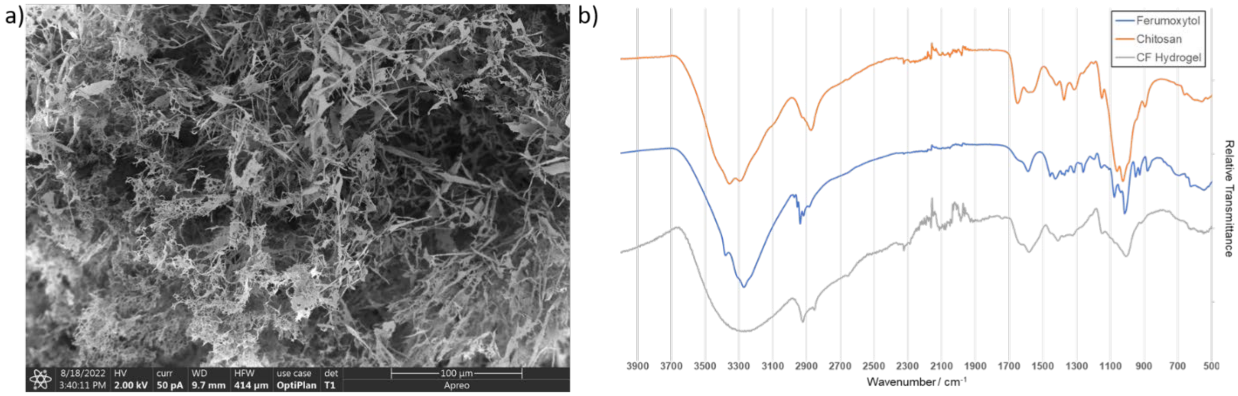

Figure S3. **a)** SEM image of the lyophilized CF hydrogel. **b)** FT-IR spectrum of ferumoxytol, chitosan and the CF hydrogel.

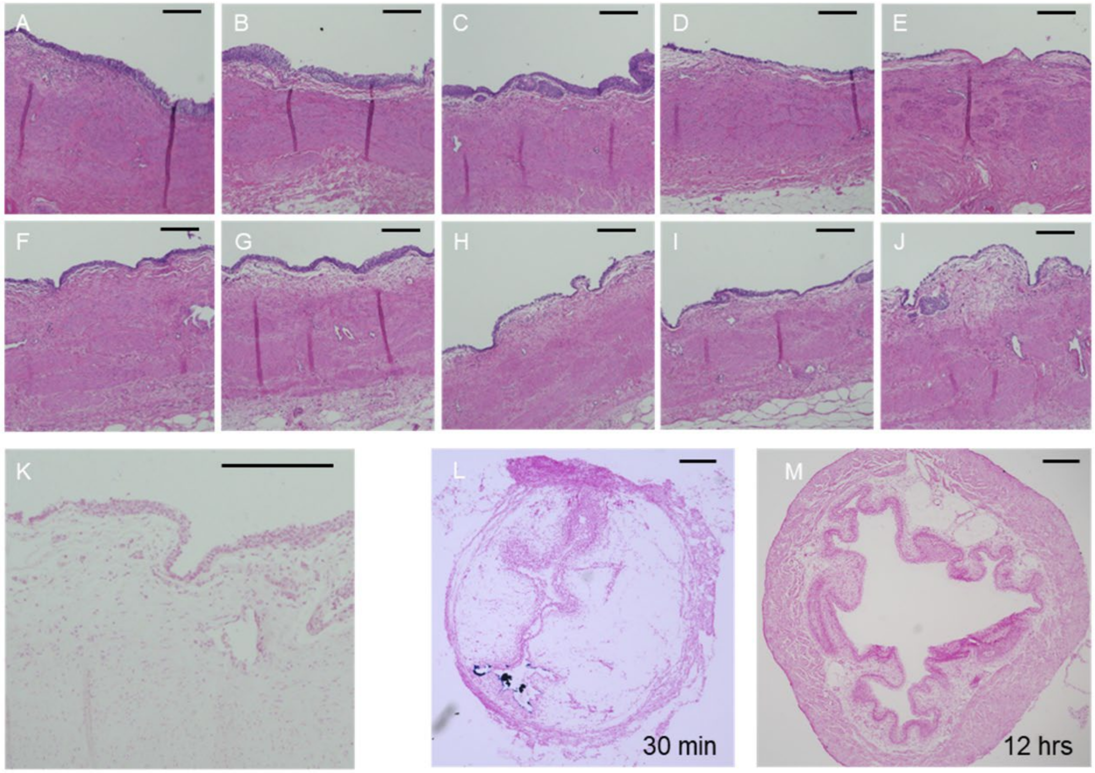

Figure S4. **A-J)** Lower magnification images of hematoxylin & eosin-stained human urothelium (Figure 4) incubated with hydrogel components *ex vivo*. Compared to normal urothelium (A), urothelium exposed to (B) 0.05% w/v chitosan for 1 minute or (C) 0.5% w/v chitosan for 1 minute did not show reductions in urothelial thickness. Urothelium exposed for longer durations to (D) 0.05% w/v chitosan for 30 minutes or (E) 0.5% w/v chitosan for 30 minutes showed significant exfoliation of the urothelial cell layers. Compared to normal urothelium (F), urothelium exposed to (G) 0.1x CF hydrogel or (H) 1x CF hydrogel for 1 minute showed minimal changes in urothelial thickness. Urothelium exposed to (I) 0.1x CF hydrogel or (J) 1x CF hydrogel for 30 minutes showed loss of superficial cell layers, though to a lower extent compared to urothelium exposed to the same time and concentration of chitosan. **K)** Prussian blue-stained human urothelium exposed to 1x CF hydrogel for 30 minutes *ex vivo*. No residual iron-containing CF hydrogel (dark blue) is seen. **L-M)** Prussian blue-stained murine bladder exposed to 1x CF hydrogel for 15 minutes. The mouse was allowed to void and was sacrificed at 30 minutes (L) or 12 hours post exposure (M). A small amount of iron-containing CF hydrogel is present within the lumen of the bladder at 30 minutes, but no hydrogel remains at 12 hours. Scale bars = 250 μm.

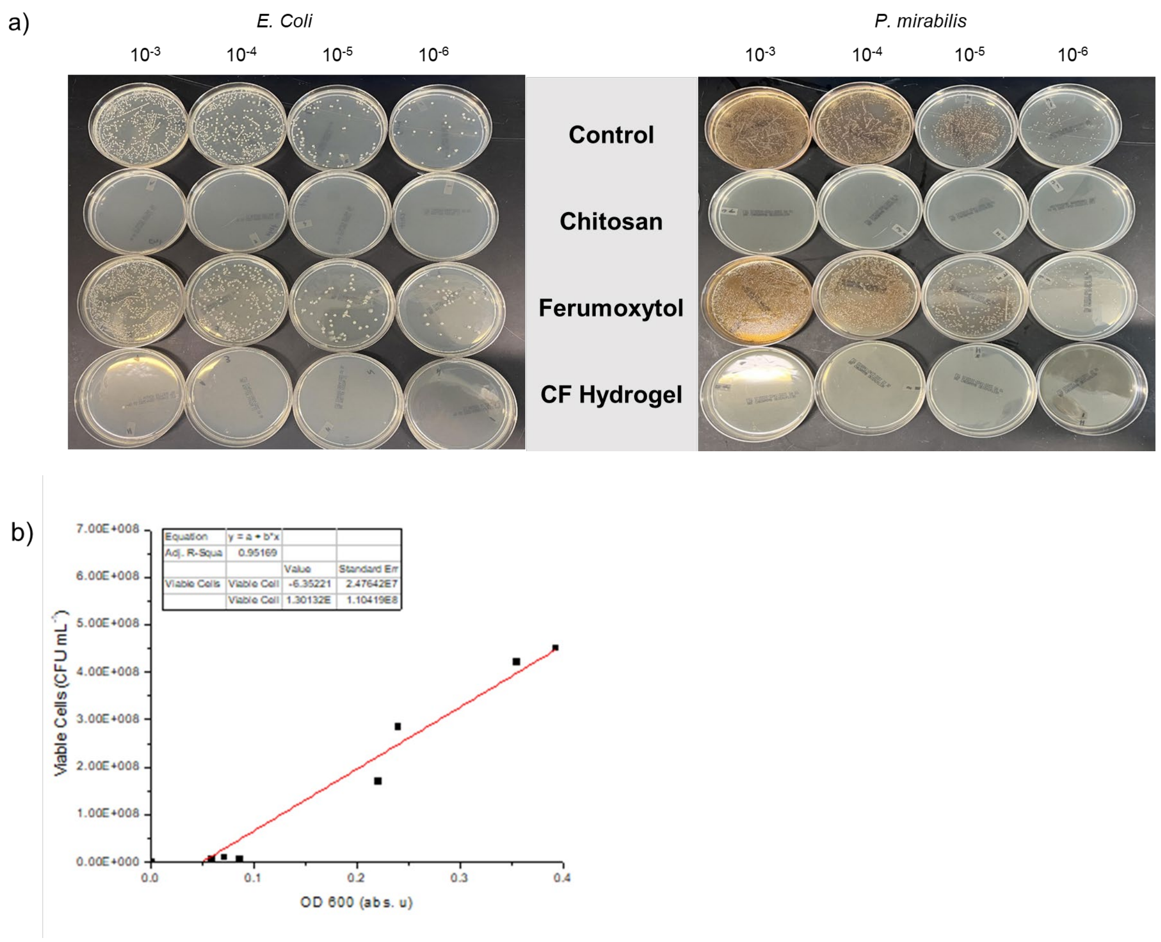

Figure S5. a) *E. coli* and *P. mirabilis* agar plates with 10-fold serial dilutions (10^{-3} to 10^{-6}) from each culture condition after 240 minutes of incubation. The plain control and ferumoxytol cultures grew to 10^8 CFU/mL while the chitosan and CF hydrogel inhibited bacterial growth. **b)** Linear relationship between OD600 and the number of viable cells (CFU/mL). Source data are provided as a Source Data file.

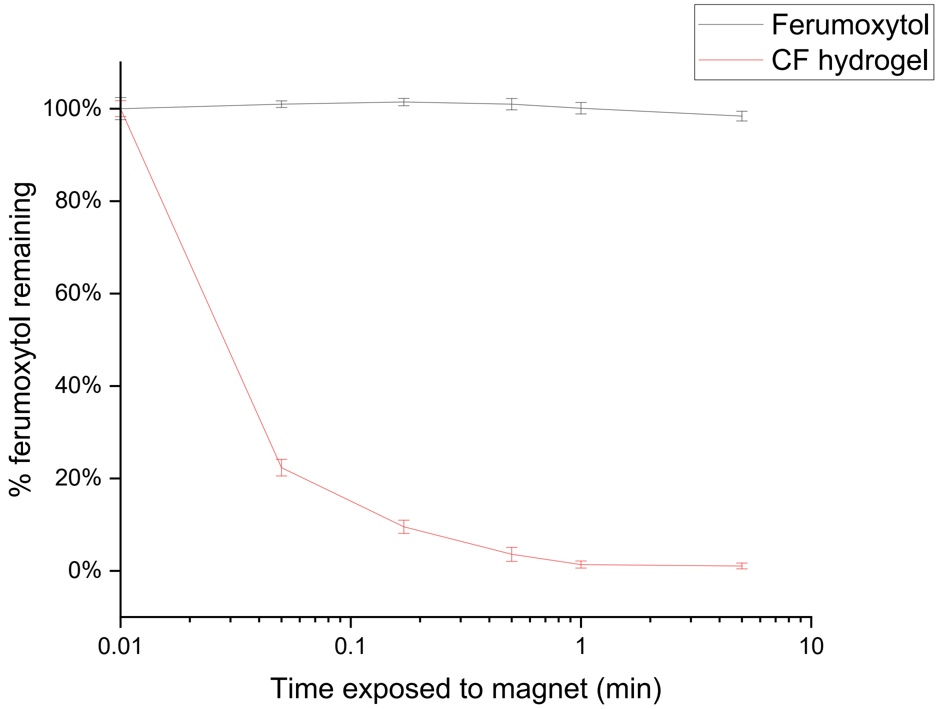

Figure S6. Excess CF hydrogel is rapidly cleared from solution by the magnetic wire. While ferumoxytol alone is not magnetically separated as the magnetophoretic motion of a small (7 nm) particle is outweighed by Brownian motion and drag, CF hydrogel forms larger aggregates of ferumoxytol particles which are quickly captured by the magnet. > 90% of CF hydrogel is cleared within the first ten seconds of exposure to the magnet, and ~99% is cleared by 1 minute of exposure. n = 3 independent experiments for each group. Data represents mean ± standard deviation. Source data are provided as a Source Data file.

Supplemental Notes

1 Magnetophoretic force

The force on a magnetic particle is

$$F = \frac{V\chi}{\mu_0}(\vec{\mathbf{B}} \cdot \nabla)\vec{\mathbf{B}} \tag{1}$$

where V is the volume of the particle, χ is the magnetic susceptibility of the particle (assuming the magnetic susceptibility of the surrounding buffer is zero), $\mu_0 = 4\pi \times 10^{-7}$ (T m A^{-1}), and B is the magnetic field (T). Here we are also assuming that the particles exhibit magnetic saturation. For SPIONs like ferumoxytol, these saturate on the order of millitesla, and our magnetic fields at the relevant distances are on the order of 0.5 T.

The measured magnetic susceptibilities of the superparamagnetic particles are mass susceptibilities χ_m, given in units of m^3 kg^{-1}, so we multiply them by their density ρ.

$$\begin{aligned}
\vec{\mathbf{B}} \cdot \nabla &= \begin{bmatrix} B_x \\ B_y \\ B_z \end{bmatrix} \cdot \begin{bmatrix} \frac{\partial}{\partial x} & \frac{\partial}{\partial y} & \frac{\partial}{\partial z} \end{bmatrix} \\
&= B_x \frac{\partial}{\partial x} + B_y \frac{\partial}{\partial y} + B_z \frac{\partial}{\partial z} \\
(\vec{\mathbf{B}} \cdot \nabla)\vec{\mathbf{B}} &= \left(B_x \frac{\partial}{\partial x} + B_y \frac{\partial}{\partial y} + B_z \frac{\partial}{\partial z} \right) \begin{bmatrix} B_x \\ B_y \\ B_z \end{bmatrix} \\
&= \begin{bmatrix} B_x \frac{\partial B_x}{\partial x} + B_y \frac{\partial B_x}{\partial y} + B_z \frac{\partial B_x}{\partial z} \\ B_x \frac{\partial B_y}{\partial x} + B_y \frac{\partial B_y}{\partial y} + B_z \frac{\partial B_y}{\partial z} \\ B_x \frac{\partial B_z}{\partial x} + B_y \frac{\partial B_z}{\partial y} + B_z \frac{\partial B_z}{\partial z} \end{bmatrix}
\end{aligned} \tag{2}$$

Therefore the magnetophoretic force in the x direction is

$$F_{m,x} \propto \left(B_x \frac{\partial B_x}{\partial x} + B_y \frac{\partial B_x}{\partial y} + B_z \frac{\partial B_x}{\partial z} \right). \tag{3}$$

2 Magnetic field gradient

The magnetic field gradient ∇B is

$$\nabla B = \begin{bmatrix} \frac{\partial B_x}{\partial x} & \frac{\partial B_y}{\partial x} & \frac{\partial B_z}{\partial x} \\ \frac{\partial B_x}{\partial y} & \frac{\partial B_y}{\partial y} & \frac{\partial B_z}{\partial y} \\ \frac{\partial B_x}{\partial z} & \frac{\partial B_y}{\partial z} & \frac{\partial B_z}{\partial z} \end{bmatrix} \tag{4}$$

The magnitude of the magnetic field gradient is given by the Frobenius norm

$$\|\nabla B\| = \sqrt{\left(\frac{\partial B_x}{\partial x}\right)^2 + \left(\frac{\partial B_y}{\partial x}\right)^2 + \left(\frac{\partial B_z}{\partial x}\right)^2 + \left(\frac{\partial B_x}{\partial y}\right)^2 + \left(\frac{\partial B_y}{\partial y}\right)^2 + \left(\frac{\partial B_z}{\partial y}\right)^2 + \left(\frac{\partial B_x}{\partial z}\right)^2 + \left(\frac{\partial B_y}{\partial z}\right)^2 + \left(\frac{\partial B_z}{\partial z}\right)^2} \tag{5}$$
