# Supplementary material for: A magnetic hydrogel for the efficient retrieval of kidney stone fragments during ureteroscopy
Source: Nat Commun. 2023 Jun 22;14:3711. doi: 10.1038/s41467-023-38936-1 (PMC10287666; doi:10.1038/s41467-023-38936-1)
Supplement: Supplementary file 3 — Description of Additional Supplementary Files [file 41467_2023_38936_MOESM3_ESM.pdf]

Description of Additional Supplementary Files

Supplementary Movie 1. A cross-section of the magnetic wire is shown with radial magnetization in the vertical axis. The magnetic gradient exerted by the wire is shown in the red-blue logarithmic color scale, showing that the gradients are stronger in the vertical axis compared to the horizontal axis. Gravitational force is not simulated, as the particles (1 mm diameter magnetically labeled stone fragments) are assumed to be settled on the floor of the kidney, thus the only active forces are magnetophoresis and drag. Particle motion is simulated over 2 seconds. Particles from up to 3.5 mm away in the vertical axis experience a net attractive magnetophoretic force and are drawn towards the magnet. Once the particle reaches within 1-2 mm of the wire, it experiences exponentially higher magnetic gradients and is quickly (in < 0.2 seconds) captured on the wire surface.

Supplementary Movie 2. The simulation from Supplementary Movie 1 is repeated with particles representing magnetically labeled stone fragments 3 mm in diameter, instead of 1 mm in diameter. Again, gravitational force is not simulated, as the stone fragments are assumed to be settled on the floor of the kidney, thus the only active forces are magnetophoresis and drag. Magnetic capture is similar, where particles from up to 3 mm away in the vertical axis experience a net attractive magnetophoretic force and are drawn towards the magnet.
