# Supplementary material for: A magnetic hydrogel for the efficient retrieval of kidney stone fragments during ureteroscopy
Source: Nat Commun. 2023 Jun 22;14:3711. doi: 10.1038/s41467-023-38936-1 (PMC10287666; doi:10.1038/s41467-023-38936-1)
Supplement: Supplementary file 6 — Reporting Summary [file 41467_2023_38936_MOESM6_ESM.pdf]

nature portfolio

Corresponding author(s): Joseph Liao

Last updated by author(s): May 6, 2023

Reporting Summary

Nature Portfolio wishes to improve the reproducibility of the work that we publish. This form provides structure for consistency and transparency in reporting. For further information on Nature Portfolio policies, see our Editorial Policies and the Editorial Policy Checklist.

Statistics

For all statistical analyses, confirm that the following items are present in the figure legend, table legend, main text, or Methods section.

n/a	Confirmed	
☐	☒	The exact sample size (*n*) for each experimental group/condition, given as a discrete number and unit of measurement
☐	☒	A statement on whether measurements were taken from distinct samples or whether the same sample was measured repeatedly
☐	☒	The statistical test(s) used AND whether they are one- or two-sided<br>*Only common tests should be described solely by name; describe more complex techniques in the Methods section.*
☒	☐	A description of all covariates tested
☒	☐	A description of any assumptions or corrections, such as tests of normality and adjustment for multiple comparisons
☐	☒	A full description of the statistical parameters including central tendency (e.g. means) or other basic estimates (e.g. regression coefficient) AND variation (e.g. standard deviation) or associated estimates of uncertainty (e.g. confidence intervals)
☐	☒	For null hypothesis testing, the test statistic (e.g. *F*, *t*, *r*) with confidence intervals, effect sizes, degrees of freedom and *P* value noted<br>*Give P values as exact values whenever suitable.*
☒	☐	For Bayesian analysis, information on the choice of priors and Markov chain Monte Carlo settings
☒	☐	For hierarchical and complex designs, identification of the appropriate level for tests and full reporting of outcomes
☒	☐	Estimates of effect sizes (e.g. Cohen's *d*, Pearson's *r*), indicating how they were calculated

Our web collection on statistics for biologists contains articles on many of the points above.

Software and code

Policy information about availability of computer code

Data collection	Zen 2.1 software was used to obtain microscope images. COMSOL Multiphysics 5.5 was used to generate magnetic simulation data. No custom algorithms or software were used
Data analysis	Figures were generated with Origin 2021b. No custom algorithms or software were used

For manuscripts utilizing custom algorithms or software that are central to the research but not yet described in published literature, software must be made available to editors and reviewers. We strongly encourage code deposition in a community repository (e.g. GitHub). See the Nature Portfolio guidelines for submitting code & software for further information.

Data

Policy information about availability of data

All manuscripts must include a data availability statement. This statement should provide the following information, where applicable:

- Accession codes, unique identifiers, or web links for publicly available datasets
- A description of any restrictions on data availability
- For clinical datasets or third party data, please ensure that the statement adheres to our policy

Data have been made available in a public repository at https://osf.io/a843d/

Human research participants

Policy information about studies involving human research participants and Sex and Gender in Research.

Reporting on sex and gender	Findings do not apply to sex/gender and sex/gender were not considered. However, participants from which human urothelium was obtained were recruited from the VA, which has a predominantly male population and therefore all participants happened to be male.
Population characteristics	Participants from which human urothelium were obtained underwent nephrectomy. Participants were excluded if the diagnosis was urothelial carcinoma.
Recruitment	Participants were recruited by reviewing upcoming nephrectomies being performed at the Palo Alto VA, for which the diagnosis was not urothelial carcinoma.
Ethics oversight	Human research was approved by the Palo Alto Institutional Review Board, IRB # 55427

Note that full information on the approval of the study protocol must also be provided in the manuscript.

Field-specific reporting

Please select the one below that is the best fit for your research. If you are not sure, read the appropriate sections before making your selection.

[x] Life sciences [ ] Behavioural & social sciences [ ] Ecological, evolutionary & environmental sciences

For a reference copy of the document with all sections, see nature.com/documents/nr-reporting-summary-flat.pdf

Life sciences study design

All studies must disclose on these points even when the disclosure is negative.

Sample size	For the cell culture studies all groups were n >= 5 over two separate experiments. For the human urothelium studies, each group had a sample size of n=3. Urothelium samples were derived from three separate patients. For the mouse studies, each group had n=1 mice.
Data exclusions	Data were not excluded.
Replication	Cell culture results were replicated with at least two separate experiments and all attempts at replication were successful. Human urothelium experiment technical replicates were analyzed together at multiple magnifications and sections, and the images most representative of the changes seen in all three replicates were selected for publication.
Randomization	Cell culture samples were not randomized. Human urothelium specimens were not randomized to experimental conditions. Experiments were designed to maximize achieving three technical replicates within a biological replicate, as the specimens were obtained on separate days with limited tissue available per day. Mice were selected randomly for the mouse bladder experiments.
Blinding	Blinding was not possible as the solutions of chitosan and ferumoxytol are visibly different colors.

Reporting for specific materials, systems and methods

We require information from authors about some types of materials, experimental systems and methods used in many studies. Here, indicate whether each material, system or method listed is relevant to your study. If you are not sure if a list item applies to your research, read the appropriate section before selecting a response.

Materials & experimental systems

n/a	Involved in the study	
[ ]	[x]	Antibodies
[ ]	[x]	Eukaryotic cell lines
[x]	[ ]	Palaeontology and archaeology
[ ]	[x]	Animals and other organisms
[x]	[ ]	Clinical data
[x]	[ ]	Dual use research of concern

Methods

n/a	Involved in the study	
[x]	[ ]	ChIP-seq
[x]	[ ]	Flow cytometry
[x]	[ ]	MRI-based neuroimaging

Antibodies

Antibodies used	Primary antibodies used were chicken anti-Krt5 (1:1000; 905901, BioLegend) and guinea pig anti-Krt20 (1:500; GP-K20, ProGen). Secondary antibodies (Alexa Fluor 633 anti-chicken, or 488 anti-guinea pig, 1:500; Molecular Probes) were diluted at 1:1000
Validation	All primary antibodies were verified by the manufacturer for activity against mice

Eukaryotic cell lines

Policy information about cell lines and Sex and Gender in Research

Cell line source(s)	ATCC - Human T24 bladder transitional cell carcinoma cells
Authentication	Authentication of cell lines was not performed beyond the initial authentication provided by ATCC
Mycoplasma contamination	Cell lines were not tested for mycoplasma contamination but no indication of contamination was observed
Commonly misidentified lines (See ICLAC register)	None

Animals and other research organisms

Policy information about studies involving animals; ARRIVE guidelines recommended for reporting animal research, and Sex and Gender in Research

Laboratory animals	Wild type mice 2-6 months old were used. They were housed with a 12-hour light dark cycle with room temperature 68-75F and humidity 45-65%
Wild animals	No wild animals were used
Reporting on sex	No findings apply to only one sex
Field-collected samples	No field collected samples
Ethics oversight	Administrative Panel on Laboratory Animal Care at Stanford University

Note that full information on the approval of the study protocol must also be provided in the manuscript.
